# The Epidemiology of Multidrug-Resistant Pathogens in Hematopoietic Stem Cell Transplantation (HSCT) Patients: A Five-Year Retrospective Study at a Cancer Center

**DOI:** 10.3390/pathogens15070684

**Published:** 2026-06-28

**Authors:** Sawsan Mubarak, Joud Jarrah, Yara K. Edor, Omar Khresat, Hadeel AlGhawrie

**Affiliations:** 1 Infection Control Program, King Hussein Cancer Center, Amman 11941, Jordan; smubarak@khcc.jo (S.M.); okhresat@khcc.jo (O.K.); 2 Department of Medicine, King Hussein Cancer Center, Amman 11941, Jordan; jj.11090@khcc.jo; 3 Department of Microbial Infection and Immunity, The Ohio State University, Columbus, OH 43210, USA; yara.edor@osumc.edu

**Keywords:** hematopoietic stem cell transplantation (HSCT), multidrug-resistant pathogens, epidemiology, antimicrobial resistance, Gram-negative bacteria, extended-spectrum beta-lactamase (ESBL), retrospective study

## Abstract

Multidrug-resistant (MDR) pathogens present a significant threat to hematopoietic stem cell transplant (HSCT) recipients; despite their critical implications, regional data on their infection patterns remain scarce. This study aimed to characterize the incidence, pathogen and antimicrobial resistance distribution of clinically confirmed bacterial infections among HSCT recipients. A retrospective analysis was conducted at King Hussein Cancer Center, Jordan (2018–2022). MDR pathogens were defined per CDC criteria. During the study period, 1157 HSCT procedures were performed. A total of 327 patients developed clinically documented bacterial infections, yielding an overall cumulative incidence of 28.3%, with a higher burden in the pediatric cohort (34.7%), including exclusive identification of *Klebsiella oxytoca* in pediatrics (2.3%). Gram-negative bacteria dominated, with *Escherichia coli* (50.5%) and *Klebsiella pneumoniae* (22.0%) being most common. Extended-spectrum beta-lactamase (ESBL) production was the dominant resistance mechanism (71.3%), followed by carbapenem-resistant *Enterobacteriaceae* (CRE; 14.1%), methicillin-resistant *Staphylococcus aureus* (MRSA; 8.6%), and carbapenem-resistant *Pseudomonas aeruginosa* (CRPA; 7.0%). The urogenital (39.1%) and bloodstream (31.2%) were the most infected sites. Significant site-specific associations were noted for ESBL production, MDR-*Acinetobacter baumannii* (*p* < 0.001) and MRSA (*p* = 0.007). Temporal analysis revealed a convergent MDR peak in 2021. Our findings offer critical insights into MDR pathogen incidence in HSCT recipients in the Middle East, informing improved infection management and intensified antimicrobial stewardship in this high-risk population.

## 1. Introduction

Emerging multidrug-resistant (MDR) pathogens pose a great challenge to the treatment of infectious diseases. This mounting threat is particularly concerning amongst hematopoietic stem cell transplant (HSCT) recipients, who endure profound immunosuppression as part of their treatment [1].

Hematopoietic cell transplantation (HCT) is a curative modality commonly used to treat malignant hematological neoplasms and nonmalignant hematological disorders. The number of transplantations is on the rise, currently at over 1.4 million transplants. However, this procedure is associated with a high risk of treatment-related mortality (TRM). Infections, organ toxicity, and graft-versus-host disease (GvHD) are some of the main contributors to TRM. Among these, more than half of fatal infections after HSCT are of unspecified etiology. Bacterial infections make up about 15% of the known causes, fungi 11%, viruses 9%, parasites 1%, and infections of mixed origin account for 5%. Bacterial pathogens account for the majority of microbiologically documented infectious fatalities, capitalizing on the profound and prolonged immunosuppression, mucosal barrier disruption, and frequent use of indwelling central venous catheters inherent to the HSCT process [2,3].

Bacterial infections pose a challenge in the care of hematopoietic stem cell transplant (HSCT) recipients [3]. Infections and their complications continue to be important contributors to mortality in hematopoietic stem cell transplantation. In addition, growing antimicrobial resistance has been linked to rising mortality rates [4]. However, in the last decade, advancements in the prevention and treatment of infectious diseases have improved transplantation outcomes [5]. Moreover, HSCT recipients are particularly vulnerable to bacterial infections in response to prolonged hospitalization, immunosuppressive treatments, invasive procedures, and frequent exposure to antibiotics. Several studies have also confirmed that Gram-negative bacterial infections are strongly associated with poor prognosis [6].

Post-allogeneic HSCT, during the early pre-engraftment phase, was dominated by Gram-positive bacterial (GPB) infections, primarily driven by skin flora and catheter utilization. However, recent decades have witnessed a critical epidemiological shift toward Gram-negative bacterial (GNB) infections originating from endogenous gastrointestinal flora. This shift is compounded by a surging global crisis of antimicrobial resistance (AMR). Under normal circumstances, only 30–40% of febrile neutropenic episodes can be microbiologically documented [7]. Many HSCT centers have reported that the emergence of infections caused by MDR microorganisms, particularly GNB, has steadily led to high rates of TRM. With this, increasing antimicrobial resistance has also been linked to rising mortality rates [4].

In recent years, a reduction in the incidence of Gram-negative (GN) infections in favor of Gram-positive (GP) bacterial infections, along with higher rates of MDR infections, has been observed in south-eastern versus north-western European regions [8]. Moreover, a significant increase in antibiotic resistance has been documented worldwide; HSCT recipients are especially susceptible due to recurrent, prolonged hospitalizations and the use of both prophylactic and broad-spectrum antibiotic therapy before and after transplantation [9].

According to available but incomplete statistics, the quinoline resistance rate has increased to 86% in some countries. Additionally, the incidence of carbapenem resistance increases to 25% from the initial state of zero resistance in post-transplantation patients, on average, every year. The overall resistance rate of tigecycline—one of the last remaining antibiotic treatment options—has not yet been reported in China or abroad [10]. A study presents a single-center retrospective analysis of colonization and infection epidemiology, and in the post-transplantation period, infections occurred in 77.4% of patients after auto-HSCT. Bacteremia was observed in 43.5% of patients, mostly caused by methicillin-resistant coagulase-negative *Staphylococcus epidermidis* (MRCNSE) [2].

The epidemiology of MDR pathogens is highly dynamic and exhibits stark geographic variability. In a study conducted at an Australian HSCT center, it was found that *Staphylococcus aureus* was the most common Gram-positive microorganism, but trends to increasing coagulase-negative *staphylococci* and vancomycin-resistant *enterococci* were observed. In the same study, *Escherichia coli* was the most common Gram-negative isolate, and Carbapenem resistance was found in *pseudomonads* and *Acinetobacter* isolates [4]. Understanding the specific regional burden of MDR pathogens is the first critical step in optimizing empiric antimicrobial stewardship for immunocompromised populations.

Among these emerging threats, *Enterococcus* is now recognized as an increasingly important pathogen in HSCT recipients, with rising vancomycin resistance, noted again in a study done on the “risk factors for enterococcal bacteremia in allogeneic hematopoietic stem cell transplant recipients” [11].

Furthermore, the clinical spectrum of bacterial complications post-HSCT is diverse, primarily manifesting as bloodstream infections (BSI), followed by pneumonia and gastrointestinal complications. The most frequent pathogens were coagulase-negative *staphylococci* and *Enterobacteriaceae*, followed by *enterococci*, *Pseudomonas aeruginosa*, and viridans *streptococci* [12]. The epidemiology and the prevalence of resistant strains vary significantly between transplant centers. In some regions, MDR Gram-negative rods are increasingly frequent. In others, vancomycin-resistant enterococci are predominant [12].

Given this heterogeneous and shifting epidemiology, comprehensive screening is recommended prior to and during transplantation to identify patients at risk of certain infectious diseases and to prevent the transmission of MDR and pathogenic microorganisms. Screening procedures should assess for colonization by methicillin-resistant *Staphylococcus aureus* (MRSA), vancomycin-resistant *Enterococcus*, and MDR Gram-negative bacteria [13].

In essence, since infections pose a major threat to HSCT patients, the workup of infection starts with an understanding of the pathogens and of the spectrum of infections associated with the disease. Updates on the epidemiology of MDR pathogens are important, especially in immunocompromised populations like HSCT patients. These patients present unique challenges to healthcare systems due to their severe susceptibility to infections. However, despite the burden of MDR in HSCT patients, data regarding risk factors and outcomes of MDR bacteria in HSCT are scarce.

To our knowledge, no population-based study has been conducted to assess the prevalence, patterns, and incidence of MDR pathogens amongst HSCT in Jordan or the Middle East to date. Therefore, it is vital to evaluate the microbiology of patients post-HSCT in this region. Awareness of the risk factors for infections, the changing epidemiology, and the resistance patterns of pathogenic microorganisms is essential for proper and effective management of infections in cancer patients.

The aim of this study was to investigate and analyze the prevalence, microbial epidemiology, and incidence of MDR pathogens among patients undergoing hematopoietic stem cell transplantation over a 5-year period at a major oncology center serving patients across the Middle East, located in the capital of Jordan, Amman. By defining this landscape at the King Hussein Cancer Center, this research seeks to provide the foundational data necessary to refine institutional stewardship protocols and improve clinical outcomes in the region.

## 2. Materials and Methods

### 2.1. Design and Setting

This study is a retrospective analysis of patients (adults and pediatrics) undergoing hematopoietic stem cell transplantation (HSCT) from all donor types and stem cell sources who subsequently developed bacterial infections at the King Hussein Cancer Center (KHCC) during a period spanning from January 2018 to December 2022. The study was approved by KHCC Institutional Review Board (IRB) (23 KHCC 132) which ensured ethical compliance and oversight.

### 2.2. Patient Population and Data Collection

The study targeted all patients who underwent either autologous or allogeneic HSCT at KHCC and subsequently developed a bacterial infection. The incidence of infection was defined as the number of patients developing at least one confirmed clinical bacterial infection divided by the total number of HSCT recipients during the same calendar year.

Patients were identified through the institutional electronic health record (EHR) system, and all positive cultures were cross-referenced with clinical symptoms to strictly exclude colonization cases, as per the established definition of infection. While routine surveillance screening for multidrug-resistant organism (MDRO) colonization—including rectal, nasal and throat swabs—were strictly excluded from the current analysis. Only isolates obtained from clinical specimens associated with symptomatic infection (e.g., blood, urine, respiratory tract, or wound cultures) were included in the study. To prevent data duplication, we applied the ‘one patient, one isolate’ rule; specifically, only the first unique bacterial species isolated from a specific clinical site per infectious episode was included in the analysis. Subsequent identical isolates from the same patient during the same episode were excluded.

A total of 327 patients met the inclusion criteria and were included in the final analysis. The clinical and microbiological variables collected for each patient included incidence, year of infection, patient age, specimen type, isolated bacterial species, and the corresponding antimicrobial resistance profiles.

### 2.3. Definitions and Clinical Management

In this study, a clear distinction was maintained between clinical infection isolates and colonization surveillance. Clinical infection isolates were defined as those recovered from diagnostic specimens—including blood, respiratory tract, urinary tract, and skin or soft tissue—where the patient exhibited clinical signs and symptoms of infection according to the Centers for Disease Control and Prevention (CDC) National Healthcare Safety Network (NHSN) criteria. Conversely, colonization surveillance isolates, obtained via routine screening protocols (such as rectal and nasal swabs), were strictly excluded from the epidemiological analysis to ensure the results represent true clinical infections rather than asymptomatic carriage [14].

Clinical specimens were collected upon the first clinical suspicion of infection (e.g., fever, localized symptoms) according to institutional standard operating procedures. For suspected bloodstream infections, at least two sets of blood cultures (one aerobic and one anaerobic bottle per set) were collected from different sites or via central and peripheral access to ensure diagnostic accuracy and exclude contamination. Urinary tract infection (UTI) samples were obtained via mid-stream clean catch or catheterization, while respiratory tract infection (RTI) samples included sputum or bronchoalveolar lavage.

Microorganism identification and antimicrobial susceptibility testing (AST) were performed using the VITEK^®^ 2 automated system (bioMérieux, Marcy-l’Étoile, France) and MALDI-TOF MS (Bruker Daltonics, Bremen, Germany). All susceptibility results were interpreted according to the Clinical and Laboratory Standards Institute (CLSI) M100 guidelines applicable at the time of isolation. Multidrug-resistant (MDR) isolate; the CDC typically uses this term to refer to an isolate that is resistant to at least one antibiotic in three or more drug classes [15]. MDR-bacteria were defined according to CDC definitions as follows [16]:

- MRSA: Includes *Staphylococcus aureus* cultured from any specimen that tests oxacillin-resistant or cefoxitin-resistant by standard susceptibility testing methods.
- VRE: Any *Enterococcus* spp. (regardless of whether identified to the species level) that is resistant to vancomycin by standard susceptibility testing methods.
- CRE: Any *Enterobacteriaceae* spp. (*Escherichia coli*, *Klebsiella oxytoca*, *Klebsiella pneumoniae* or *Enterobacter* spp.) testing non-susceptible (i.e., resistant or intermediate) to imipenem or meropenem by standard susceptibility testing methods, or with a positive result for any FDA-approved method for carbapenemase detection from specific specimen sources; and resistant to all third-generation cephalosporins tested.
- CRPA: *Pseudomonas aeruginosa* exhibiting non-susceptibility (intermediate or resistant) to imipenem or meropenem, corresponding to a Minimum Inhibitory Concentration (MIC) more than 8 μg/mL per CLSI M100 guidelines.
- MDR-*Acinetobacter*: any *Acinetobacter* spp. testing non-susceptible (i.e., resistant or intermediate) to imipenem and meropenem by standard susceptibility testing methods, or with a positive result for any FDA-approved method for carbapenemase detection from specific specimen sources; and resistant to all third-generation cephalosporins tested.

### 2.4. Statistical Analysis

IBM SPSS Statistics for Windows, Version 27.0 (IBM Corp., Armonk, NY, USA) was used to analyze the data. Categorical variables were reported as frequency counts and percentages, whereas continuous variables were reported as mean and standard deviation. Moreover, a cross-tabulation analysis using Pearson’s chi-square (χ2) test (Fisher’s exact test was used for cells with expected frequency < 5) was conducted to examine significant differences between categorical variables. Logistic regression was used to compute the adjusted odds ratio (95% CI) and to investigate the relationship between binary response variables and a set of explanatory, or independent, variables. T-test and ANOVA were used to examine significant differences between continuous variables. Temporal trends in the prevalence of antimicrobial resistance phenotypes were evaluated using a two-staged approach. First, descriptive annual prevalence was calculated as the proportion of specific resistance phenotypes among the total number of clinical bacterial infections confirmed within each calendar year. Second, to determine the statistical significance of these fluctuations, a multivariable logistic regression model was employed. In this model, the year of infection was treated as a continuous independent variable to calculate the trend coefficient, while simultaneously adjusting for patient age and sex as potential confounders.

### 2.5. Data Availability

The datasets generated and analyzed during the current study are available from the corresponding author upon reasonable request.

## 3. Results

### 3.1. Incidence and Baseline Characteristics

Over the five-year retrospective period from 2018 to 2022, a total of 1157 HSCT procedures were performed at our center, comprising 774 adult and 383 pediatric recipients. Among these, 327 patients developed clinical bacterial infections, resulting in an overall infection incidence of 28.3%, the annual incidence fluctuated during the study period, peaking at 41.2% in 2021. The 2021 overall incidence figure (41.2%) is now accompanied by its formal trend coefficient (β = +0.38, 95% CI: +0.14 to +0.62, *p* = 0.003) and explicitly described as a statistically significant single-year surge within a non-linear five-year pattern rather than a sustained directional trend. Stratification by age group revealed that the incidence was notably higher in the pediatric cohort (34.7%, 133/383) compared to the adult cohort (25.1%, 194/774). These results are presented in Table 1.

As detailed in Table 2, of the 327 patients with confirmed infections, 197 (60.2%) were female and 130 (39.8%) were male. The cohort comprised 194 adults (59.3%; mean age 43.36 ± 13.68 years; range 21.7–71.8 years) and 133 pediatric patients (40.7%; mean age 14.77 ± 8.03 years; range 1.6–36.4 years) (Table 3).

### 3.2. Microbiological Distribution and Resistance Profiling

Infections were predominantly localized to the urogenital system (39.1%, n = 128) and bloodstream (31.2%, n = 102), followed by the respiratory system (11.6%, n = 38), abdominal and pelvic region infections (8.0%, n = 26), and musculoskeletal infections (7.0%, n = 23).

Among the identified pathogens causing bacterial infections post-HSCT, Gram-negative bacteria were predominant, accounting for more than 85.0% of infections. *Escherichia coli* was the most prevalent, accounting for 165 cases (50.5%), followed by *Klebsiella pneumoniae*, responsible for 72 cases (22.0%), followed by *Staphylococcus aureus* with 28 cases (8.6%). Other isolated microorganisms included *Pseudomonas aeruginosa* (21 cases, 6.4%), *Enterococcus faecium* (19 cases, 5.8%), and *Acinetobacter baumannii* (19 cases, 5.8%). *Klebsiella oxytoca* was the least frequently isolated, with only 3 cases recorded (0.9%) (Table 2).

Among the pathogens, a notable percentage exhibited MDR or specific resistance patterns. Extended-spectrum beta-lactamase (ESBL) production was the most prevalent resistance pattern, observed in 71.3% (233/327) of cases. Carbapenem-resistant *Enterobacteriaceae* (CRE) were identified in 46 cases (14.1%), methicillin-resistant *Staphylococcus aureus* (MRSA) in 28 cases (8.6%), carbapenem-resistant *Pseudomonas aeruginosa* (CRPA) in 23 cases (7.0%), vancomycin-resistant *enterococci* (VRE) in 17 cases, and 19 cases were characterized as MDR-*Acinetobacter baumannii*. These results are presented in Table 2.

### 3.3. Predominant Microorganism and Resistant Patterns Among HSCT Patients by Age Group

The analysis of bacterial pathogens and resistance patterns among hematopoietic stem cell transplant (HSCT) patients revealed significant microbiological divergence was observed between age cohorts (Table 4).

Among adult patients, *Escherichia coli* was the most predominant pathogen (58.2%), followed by *Klebsiella pneumoniae* (18.6%) and *Staphylococcus aureus* (6.2%). In contrast, pediatric patients exhibited a higher prevalence of *Klebsiella pneumoniae* (27.1%), followed by *Escherichia coli* (39.1%) and *Staphylococcus aureus* (12%). Notably, *Klebsiella oxytoca* was exclusively identified in the pediatric age group (2.3%).

Methicillin-resistant *Staphylococcus aureus* (MRSA) was detected in 12 (6.2%) adult patients and 16 (12%) pediatric patients. Multidrug-resistant *Acinetobacter baumannii* was observed in 11 (5.7%) adult patients and 8 (6%) pediatric patients, with no significant difference between the age groups (*p* = 0.896). Vancomycin-resistant *enterococci* (VRE) were found in 7 (3.6%) adult patients and 10 (7.5%) pediatric patients. Extended-spectrum beta-lactamase (ESBL) production was identified in 145 (74.7%) adult patients and 88 (66.2%) pediatric patients, showing a trend toward significance (*p* = 0.092). Carbapenem-resistant *Enterobacteriaceae* (CRE) were detected in 31 (16%) adult patients and 15 (11.3%) pediatric patients, with no significant difference between the age groups (*p* = 0.260). Carbapenem-resistant *Pseudomonas aeruginosa* (CRPA) was present in 15 (7.7%) adult patients and 8 (6%) pediatric patients, showing no significant difference (*p* = 0.551).

These findings underscore the importance of age-specific considerations in understanding the epidemiology and antimicrobial resistance profiles among HSCT patients, which can aid in optimizing treatment strategies and infection control measures.

### 3.4. Site of Infection Analysis

*Escherichia coli* was the most prevalent microorganism across all infection sites, with particularly high proportions observed in blood and urogenital system infections. *Klebsiella pneumoniae* and *Pseudomonas aeruginosa* were also commonly identified, although with variable prevalence across infection sites. Notably, *Acinetobacter baumannii* exhibited a higher prevalence in respiratory infections compared to other sites (Table 5).

Regarding the distribution and association of resistant patterns according to the anatomical site of infection among HSCT patients (Table 6), it was found that significant associations were observed with the distribution for MRSA varying significantly across infection sites (*p* = 0.007), and highly significant association noted in the distribution of multidrug-resistant organisms *Acinetobacter baumannii* (*p* < 0.001) and extended-spectrum beta-lactamase (ESBL) production (*p* < 0.001) across various sites. CRE and CRPA were relatively rare but were observed across multiple infection sites. These findings underscore the importance of considering infection sites when assessing microbiological profiles and resistance patterns among HSCT patients, highlighting the need for tailored treatment and infection control strategies.

### 3.5. Temporal Trends Assessment

**Figure 1:**
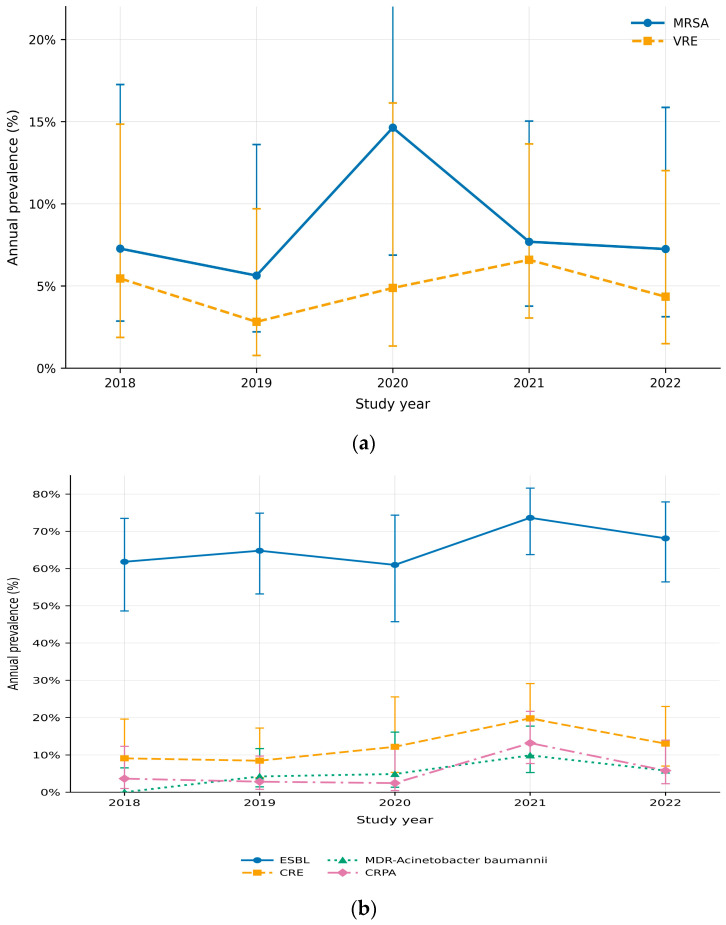
Temporal trends assessment of resistant patterns among Hematopoietic Stem Cell Transplant (HSCT) patients: (**a**) Gram-positive MDR isolates; (**b**) Gram-negative MDR isolates.

The temporal trends analysis focused on examining the prevalence of multidrug-resistant (MDR) pathogens among Hematopoietic Stem Cell Transplant (HSCT) patients over a five-year period, from 2018 to 2022, as shown in Figure 1. The analysis encompassed MRSA, MDR *Acinetobacter baumannii* organisms, VRE, CRE, ESBL producers, and CRPA. Across the study period, ESBL producers consistently exhibited the highest prevalence, with a peak of 73.6% in 2021 and a subsequent decline to 68.1% in 2022. Conversely, MDR-*Acinetobacter baumannii* organisms showed variable prevalence, ranging from absent in 2018 to 9.9% in 2021. CRE exhibited notable fluctuations, reaching a peak of 19.8% in 2021. CRPA prevalence showed minor variability, ranging from 2.2% to 13% across the study period.

Notably, MRSA prevalence fluctuated between 5.6% in 2019 and 14.6% in 2020, while VRE prevalence remained relatively stable over the years, ranging from 2.8% to 7.3%. To account for potential confounding variables, a multivariate logistic regression analysis was performed adjusting for age and sex. The analysis indicated that the temporal fluctuations observed in MRSA and VRE prevalence remained independent of these demographic factors (*p* > 0.05).

## 4. Discussion

This five-year retrospective cohort study provides the first comprehensive epidemiological characterization of multidrug-resistant (MDR) bacterial infections among hematopoietic stem cell transplant (HSCT) recipients in Jordan and, to our knowledge, the broader Middle East. By anchoring the analysis to a clearly defined denominator of 1157 HSCT procedures performed at a single high-volume oncology center, we were able to accurately quantify the true burden of clinically confirmed bacterial infections, yielding an overall cumulative incidence of 28.3%. This figure is notably higher than the 15–20% rates reported in earlier North American and European cohort studies [17,18].

One of the most clinically meaningful findings of this study is the significantly higher infection incidence observed in pediatric HSCT recipients (34.7%) compared to adults (25.1%) underscores the distinct clinical vulnerabilities inherent to pediatric transplantation and clinical challenges in managing these patients. Furthermore, the exclusive identification of *Klebsiella oxytoca* in the pediatric cohort (2.3%)—absent entirely from adult patients—points to a pathogen-niche differentiation that may warrant age-specific empiric therapy algorithms.

Considering the sharp increase in the infection incidence observed in 2021 (41.2%), it is highly probable that this peak does not represent a biological trend, but rather a reflection of external operational variables, including shifts in annual transplant volume (which dropped in 2020 and rebounded in 2021) and the systemic healthcare disruptions, altered staffing models, and changes in patient acuity driven by the COVID-19 pandemic. Similar pandemic-associated surges in healthcare-associated infections have been documented across multiple intensive care and oncology settings [19]. Having established these baseline incidence rates, our subsequent analysis of pathogen distribution and resistance patterns provides a highly contextualized view of the microbiological landscape.

Advances made in the field of hematopoietic stem cell transplantations (HSCT) over the past 20 years were predominantly driven by Gram-positive organisms, largely attributed to the widespread use of indwelling central venous catheters and mucosal barrier breakdown [14]. Studies by Krawiec et al. and Gil et al., for instance, reported that Gram-positive bacteria, particularly coagulase-negative staphylococci (CoNS), accounted for the vast majority (up to 82.9%) of all bacterial infections [2,20]. In stark contrast, our study reveals a profound epidemiological inversion: over 85% of the identified clinical infections in our cohort were caused by Gram-negative bacteria, while Gram-positive isolates accounted for merely 14%.

This massive shift toward Gram-negative dominance highlights the dynamic nature of infectious disease epidemiology in modern transplant settings. Our findings, however, are consistent with more recent global observations, such as those by Mendes et al., who noted a rising trajectory of Gram-negative infections reaching 53.8% in their cohorts [21], while Macesic et al. reported a progressive increase in Gram-negative resistance over a nine-year period in an Australian transplant center [4]. The near-exclusive dominance of Gram-negative pathogens in our center—spearheaded by *Escherichia coli* (50.5%) and *Klebsiella pneumoniae* (22.0%)—together accounting for nearly three-quarters of all isolates, likely reflects the high community prevalence of ESBL-producing *Enterobacteriaceae* in Jordan and the broader region, as well as the disruption of gastrointestinal colonization resistance by broad-spectrum antibiotic prophylaxis, and the increasing use of fluoroquinolone prophylaxis—which selects against Gram-positive flora while permitting Gram-negative overgrowth—all likely contribute.

The emergence of multidrug-resistant (MDR) pathogens within this Gram-negative shift represents a critical threat to HSCT recipients, directly correlating with increased morbidity and mortality [3]. While some institutional reports have noted a historical decline in MDR rates due to improved supportive care, earlier engraftment, and enhanced hand hygiene protocols [10], our data indicates that this progress remains fragile, particularly given the substantial elevation of MDR incidence.

ESBL production emerged as the foremost resistance mechanism in our cohort, identified in 71.3% (233/327) of all isolates and representing the single greatest antimicrobial stewardship challenge at our institution. The pronounced site-specific concentration of ESBL-producing organisms in urogenital infections (86.7%, 111/128) and abdominal/pelvic infections (65.4%, 17/26), juxtaposed with a notably lower prevalence in respiratory infections (31.6%, 12/38), likely reflects the endogenous origin of these pathogens from gastrointestinal and urinary reservoirs—consistent with the hypothesis that gut microbiome disruption during conditioning and transplantation serves as the primary seeding source for ESBL-producing *Enterobacteriaceae*.

Carbapenem-resistant *Enterobacteriaceae* (CRE) were identified in 14.1% (46/327) of patients, with a marked peak of 19.8% in 2021. This trajectory is deeply concerning from a therapeutic standpoint, as carbapenems represent the last reliable line of empiric therapy for ESBL-producing organisms, and their loss of efficacy creates a clinical scenario with severely limited options. Carbapenem-resistant *Pseudomonas aeruginosa* (CRPA) was present in 7.0% of patients (23/327), with a disproportionate concentration in respiratory infections (15.8%) and a temporal peak of 13.2% in 2021—a pattern consistent with the known tropism of *Pseudomonas aeruginosa* for the respiratory tract in immunocompromised hosts and its capacity for nosocomial amplification during periods of heightened healthcare system pressure [7].

MDR-*Acinetobacter baumannii* demonstrated a highly site-specific distribution, with 26.3% (10/38) of respiratory infection isolates carrying the MDR phenotype—a prevalence dramatically higher than at any other site. This organism’s well-documented capacity for environmental persistence, nosocomial transmission, and acquisition of pan-drug resistance mechanisms makes it a particular concern in the context of intensive care unit crossover for critically ill HSCT recipients [12]. Its emergence from zero cases in 2018 to a peak prevalence of 9.9% in 2021 is a sentinel finding that warrants dedicated infection control investigation, including environmental sampling and enhanced contact precaution protocols.

Furthermore, the resistance crisis extends to the Gram-positive spectrum; notably, methicillin-resistant *Staphylococcus aureus* (MRSA), which demonstrated a statistically significant association with infection site (*p* = 0.007), with the highest positivity rates in respiratory (15.8%) and bloodstream (10.8%) infections. When compared to the literature, our overall bacteremia rates in HSCT patients mirror the 20% to 31% prevalence described by Wang et al. [18] and Salazar et al. [17], though slightly lower than the 43.5% reported by Krawiec et al. [2]. However, the specific microbial etiology driving our bacteremia cases dictates a completely different empiric approach.

Notably, *Enterococcus faecium* was identified in 5.8% of patients, with VRE accounting for 5.2% of the total cohort—a prevalence consistent with the rising global trend of vancomycin resistance in Enterococcus among HSCT recipients, as documented by Mikulska et al. [12].

The temporal analysis reveals that MDR pathogen epidemiology in our HSCT program is not static but deeply dynamic, with meaningful inter-annual fluctuations across all six resistance phenotypes. ESBL producers consistently dominated across all five years, never falling below 61.8%, a finding that strongly supports consideration of ESBL-active empiric regimens within institutional stewardship discussions for febrile neutropenia in this population, pending prospective validation and outcome-linked assessment. The convergent 2021 peaks across ESBL, CRE, MDR-*Acinetobacter*, and CRPA reinforce the importance of interpreting point-prevalence data within its operational context, and argue strongly for prospective, real-time resistance surveillance systems that can detect emerging clusters independent of annual reporting cycles.

This variance underscores the dynamic nature of infectious disease epidemiology in transplant settings, potentially influenced by factors such as geographic location, patient demographics, antimicrobial stewardship practices, and evolving pathogen resistance patterns. These results could reflect the high rate of infection due to Gram-negative microorganisms in our hospital and could be useful to address measures to prevent and control such infections.

While this study provides foundational epidemiological data, several limitations must be acknowledged. Primarily, the retrospective, single-center design restricts the generalizability of our findings to institutions with different regional resistance patterns and clinical protocols. As the data were primarily sourced from an infection control and microbiological surveillance database, these clinical variables were not captured at the patient level. Consequently, we were unable to adjust for specific transplant subtypes (donor source and stem cell origin), variations in conditioning regimen intensity, or the exact duration of profound neutropenia. Additionally, the absence of data regarding prior intensive care unit (ICU) admission history, duration of central venous catheterization, and detailed prior antibiotic exposure prevents us from establishing independent risk factors for these infections.

Therefore, this study does not aim to serve as a predictive risk model, but rather as a comprehensive epidemiological description of the pathogens currently challenging modern transplant programs. These findings underscore the absolute necessity of continuously mapping local resistance patterns, as historical data or textbook guidelines may no longer reflect the reality of the pathogens threatening HSCT patients. Moving forward, prospective studies utilizing integrated clinical registries are essential to correlate specific transplant factors with the risk of MDR infections, ultimately guiding the optimization of empiric antimicrobial stewardship in this highly vulnerable population.

## 5. Conclusions

This study delineates the evolving epidemiology of multidrug-resistant (MDR) pathogens in hematopoietic stem cell transplant (HSCT) recipients, revealing a 28.3% overall incidence of clinical bacterial infections among HSCT recipients, with a pronounced vulnerability in the pediatric population (34.7%). Our findings demonstrate a decisive epidemiological shift toward Gram-negative bacteria as the predominant culprits of post-transplant infections, with *Escherichia coli* and *Klebsiella pneumoniae* constituting the majority of isolates. Extended-spectrum beta-lactamase (ESBL) production emerged as the foremost resistance mechanism in 71.3% of cases, reflecting a critical challenge in managing antimicrobial resistance within immunocompromised populations.

As the first comprehensive regional analysis from the Middle East, this study fills a pivotal gap in understanding MDR dynamics in HSCT populations and highlights the imperative for antimicrobial stewardship programs, enhanced surveillance, and tailored empiric therapy guidelines. These findings advocate for institutional and regional strategies to mitigate resistance risks, optimize therapeutic outcomes, and reduce morbidity in this vulnerable cohort, ultimately informing global efforts to combat antimicrobial resistance in high-risk clinical settings.

The patients who will undergo HSCT in this region over the coming decade deserve nothing less than a healthcare system that has learned from this data and acted on it with urgency and precision.

## Figures and Tables

**Table 1:** Annual Incidence of Clinical Bacterial Infections among Adult and Pediatric HSCT Recipients (2018–2022).

Year	Adult Service (N = 774)			Pediatric Service (N = 383)			Overall Cohort (N = 1157)		
	HSCT (N)	Infected (n)	Incidence (%)	HSCT (N)	Infected (n)	Incidence (%)	HSCT (N)	Infected (n)	Incidence (%)
2018	162	29	17.9	76	26	34.2	238	55	23.1
2019	168	39	23.2	85	32	37.6	253	71	28.1
2020	138	15	10.9	75	26	34.7	213	41	19.2
2021	148	66	44.6	73	25	34.2	221	91	41.2
2022	158	45	28.5	74	24	32.4	232	69	29.7
Total	774	194	25.1	383	133	34.7	1157	327	28.3

**Table 2:** Descriptive Data for All Variables of Bacterial Infections among HSCT Patients (n = 327).

Variables		Frequency	Percentage
Gender	Male	130	39.8
	Female	197	60.2
	Total	327	100.0
Age Group	Adult	194	59.3
	Pediatric	133	40.7
	Total	327	100.0
Site of Infection	Abdominal and Pelvic region	26	8.0
	Blood	102	31.2
	Musculoskeletal	23	7.0
	Urogenital system	128	39.1
	Respiratory	38	11.6
	Others	10	3.1
	Total	327	100.0
Pathogen	Gram-positive Bacteria	47	14.4
	Gram-negative Bacteria	280	85.6
	Total	327	100.0
Microorganism	*Staphylococcus aureus*	28	8.6
	*Pseudomonas aeruginosa*	21	6.4
	*Klebsiella pneumoniae*	72	22.0
	*Klebsiella oxytoca*	3	.9
	*Escherichia coli*	165	50.5
	*Enterococcus faecium*	19	5.8
	*Acinetobacter baumannii*	19	5.8
	Total	327	100.0
Resistant Pattern	MRSA	28	
	MDR-*Acinetobacter baumannii*	19	
	VRE	17	
	CRE	46	
	ESBL	233	
	CRPA	23	

Percentages are not reported for resistance patterns as individual isolates may carry more than one resistance phenotype, precluding a shared denominator.

**Table 3:** The age distribution of patients.

Age Group	N	Mean	Std. Deviation	Minimum	Maximum
Adult	194	43.36	13.68	21.7	71.8
Pediatric	133	14.77	8.03	1.6	36.4
Total	327	31.73	18.30	1.6	71.8

**Table 4:** Predominant Organism and Resistant Patterns among HSCT Patients by Age Group.

Variables			Adult (N. %)	Pediatrics (N. %)	*p*-Value
Microorganism	*Staphylococcus aureus*		12, 6.2	16, 12	0.009
	*Pseudomonas aeruginosa*		13, 6.7	8, 6	
	*Klebsiella pneumoniae*		36, 18.6	36, 27.1	
	*Klebsiella oxytoca*		0	3, 2.3	
	*Escherichia coli*		113, 58.2	52, 39.1	
	*Enterococcus faecium*		9, 4.6	10, 7.5	
	*Acinetobacter baumannii*		11, 5.7	8, 6	
	Total		194	133	
Resistant Pattern			Adult (N.)	Pediatrics (N.)	
	MRSA	Positive	12	16	0.064
		Negative	182	117	
	MDR-*Acinetobacter baumannii*	Positive	11	8	0.896
		Negative	183	125	
	VRE	Positive	7	10	0.118
		Negative	187	123	
	CRE	Positive	31	15	0.260
		Negative	163	118	
	ESBL	Positive	145	88	0.092
		Negative	49	45	
	CRPA	Positive	15	8	0.551
		Negative	179	125	

**Table 5:** Distribution of microorganisms according to the site of infection among Hematopoietic Stem Cell Transplant (HSCT) patients.

	Abdominal and Pelvic Region	Blood	Musculoskeletal	Urogenital System	Respiratory	Others/General
*Enterococcus faecium*	2, 7.69%	5, 4.90%	3, 13.04%	5, 3.91%	2, 5.26%	2, 20.00%
*Escherichia coli*	12, 46.15%	59, 57.84%	10, 43.48%	77, 60.16%	4, 10.53%	3, 30.00%
*Klebsiella pneumoniae*	6, 23.08%	17, 16.67%	3, 13.04%	35, 27.34%	10, 26.32%	1, 10.00%
*Pseudomonas aeruginosa*	2, 7.69%	3, 2.94%	3, 13.04%	5, 3.91%	6, 15.79%	2, 20.00%
*Staphylococcus aureus*	3, 11.54%	11, 10.78%	3, 13.04%	3, 2.34%	6, 15.79%	2, 20.00%
*Acinetobacter baumannii*	1, 3.85%	4, 3.92%	1, 4.35%	3, 2.34%	10, 26.32%	0, 0.00%
*Klebsiella oxytoca*	0, 0.00%	3, 2.94%	0, 0.00%	0, 0.00%	0, 0.00%	0, 0.00%
Total	26	102	23	128	38	10

**Table 6:** The association of resistant patterns according to the site of infection among Hematopoietic Stem Cell Transplant (HSCT) patients.

		Abdominal and Pelvic	Blood	Musculoskeletal	Urogenital System	Respiratory	Others/ General	Total
MRSA	Negative	24	91	20	125	32	7	299
	Positive	2	11	3	3	6	3	28
	Total	26	102	23	128	38	10	327
	*p*-value	0.007						
MDR-*Acinetobacter baumannii*	Negative	25	98	22	125	28	10	308
	Positive	1	4	1	3	10	0	19
	Total	26	102	23	128	38	10	327
	*p*-value	<0.001						
VRE	Negative	23	99	20	123	36	9	310
	Positive	3	3	3	5	2	1	17
	Total	26	102	23	128	38	10	327
	*p*-value	0.224						
CRE	Negative	23	83	18	118	31	8	281
	Positive	3	19	5	10	7	2	46
	Total	26	102	23	128	38	10	327
	*p*-value	0.154						
ESBL	Negative	9	25	11	17	26	6	94
	Positive	17	77	12	111	12	4	233
	Total	26	102	23	128	38	10	327
	*p*-value	<0.001						
CRPA	Negative	24	97	20	123	32	8	304
	Positive	2	5	3	5	6	2	23
	Total	26	102	23	128	38	10	327
	*p*-value	0.053						

## Data Availability

The datasets generated and analyzed during the current study are available from the corresponding author upon reasonable request.

## Abbreviations

The following abbreviations are used in this manuscript:

HSCT	Hematopoietic stem cell transplantation
MDR	Multidrug-resistant
CDC	Centers for Disease Control and Prevention
ESBL	Extended-spectrum beta-lactamase
CRE	Carbapenem-resistant *Enterobacteriaceae*
MRSA	Methicillin-resistant *Staphylococcus aureus*
HCT	Hematopoietic cell transplantation
TRM	Treatment-related mortality
GvHD	Graft-versus-host disease
GNB	Gram-negative bacteria
GPB	Gram-positive bacteria
GN	Gram-negative
GP	Gram-positive
MRCNSE	Methicillin-resistant coagulase-negative *Staphylococcus epidermidis*
BSI	Bloodstream infections
KHCC	King Hussein Cancer Center
IRB	Institutional Review Board
VRE	Vancomycin-resistant enterococci
CRPA	Carbapenem-resistant *Pseudomonas aeruginosa*
MRCNS	Methicillin-resistant coagulase-negative *Staphylococcus*
IBM-SPSS	Statistical Package for Social Sciences
ANOVA	Analysis of Variance
